# Supplementary material for: Airborne observations reveal elevational gradient in tropical forest isoprene emissions
Source: Nat Commun. 2017 May 23;8:15541. doi: 10.1038/ncomms15541 (PMC5457511; doi:10.1038/ncomms15541)
Supplement: Supplementary Information — Supplementary Note, Supplementary Figures, and Supplementary Tables [file ncomms15541-s1.pdf]

Supplementary Note 1. GoAmazon2014/5 research campaign. The Green Ocean Amazon (GoAmazon) 2014/5 research program was an integrated field experiment in the central Amazon basin to understand how land-atmospheric processes affect tropical hydrology and climate. The Department of Energy (DOE) Gulfstream-1 (G-1) research aircraft was used to conduct multiple measurements in the air. There were 16 research flights in the wet season during Feb 15~Mar 26, and 19 research flights in the dry season during Sep 1~Oct 10, in 2014. Many flights were conducted to characterize the evolution of the air pollutant plume downwind of Manaus, Brazil. Based on the flight maneuvers and environmental factors, we selected four research flights (i.e. 20140301a, 20140314, 20140317, 20140323) in wet season and four research flights (i.e. 20140912, 20140915,20140916, 20140930) in dry season for this study to minimize the impacts from city plumes. We assume that the PTR-MS signal at m/z 69 is only isoprene but it should be noted that there are potential interferences for this mass including fragmentation of large molecule species in diesel or gasoline exhaust, furan from biomass burning, and alkene species. These impacts are expected to be minor for the relatively clean environment of the pristine Amazon forest, and we have minimized the impacts from city plumes and biomass burning by excluding the flight segments with high values of O_3, CO, NO_x and aromatics (as indicators of biomass burning and anthropogenic pollutants).

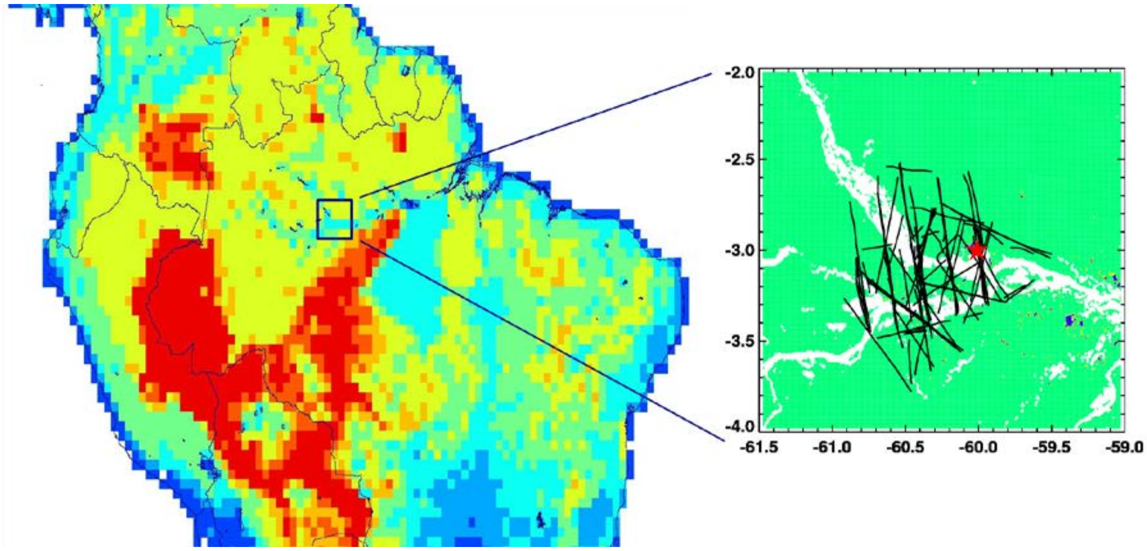

Supplementary Figure 1. Flight tracks of selected eight research flights used in this study. The city of Manaus is drawn with red star. Land-use classification in the study domain: green (trees), yellow (shrub), blue (grass or crop), and white (water).

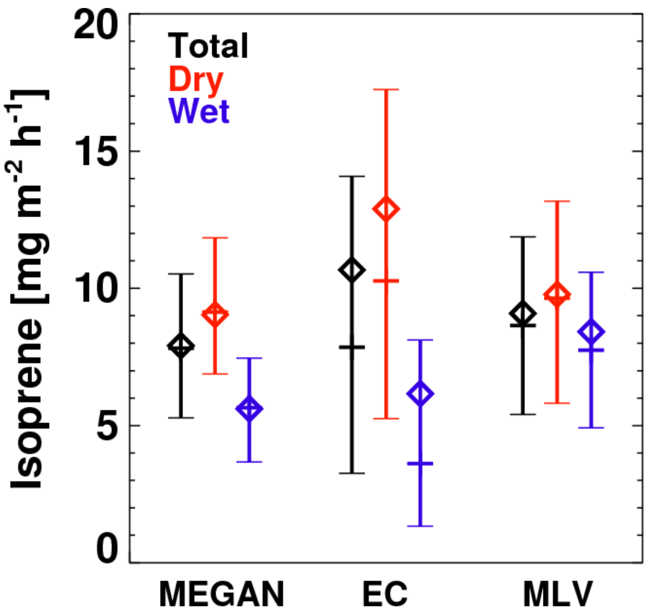

Supplementary Figure 2. Isoprene emission estimates from MLV technique. The mean values (diamond), 25% quartile values (lower bar), 50% quartile values (middle bar) and 75% quartile values (higher bar) of surface isoprene emissions from EC and MLV method compared with MEGAN for all available flights (black), dry season (red) and wet season (blue).

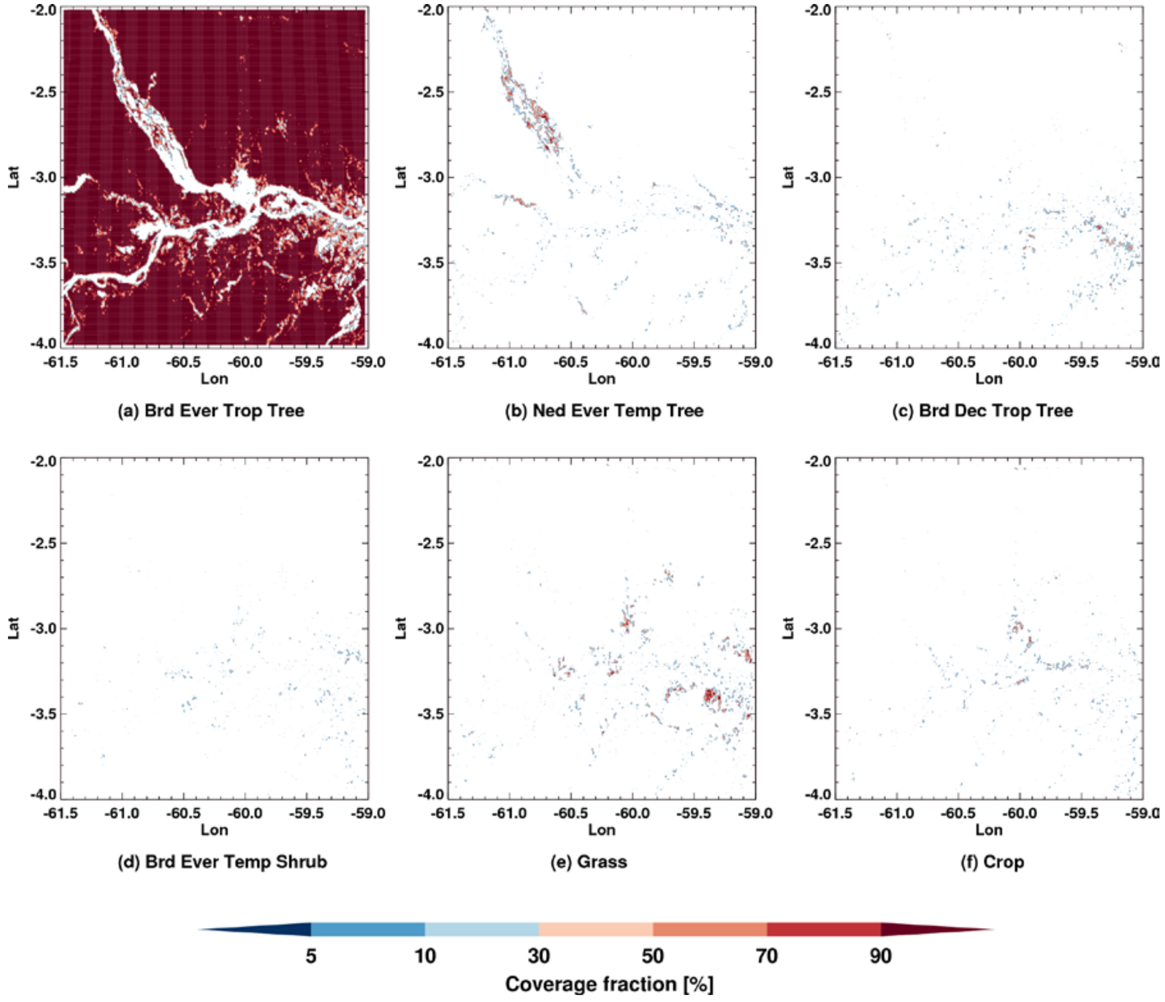

Supplementary Figure 3. **PFT coverage.** The coverage fractions of (a) broadleaf evergreen tropical tree, (b) needleleaf evergreen temperate tree, (c) broadleaf deciduous tropical tree, (d) broadleaf evergreen temperate shrub, (e) grass and (f) crop from MODIS data.

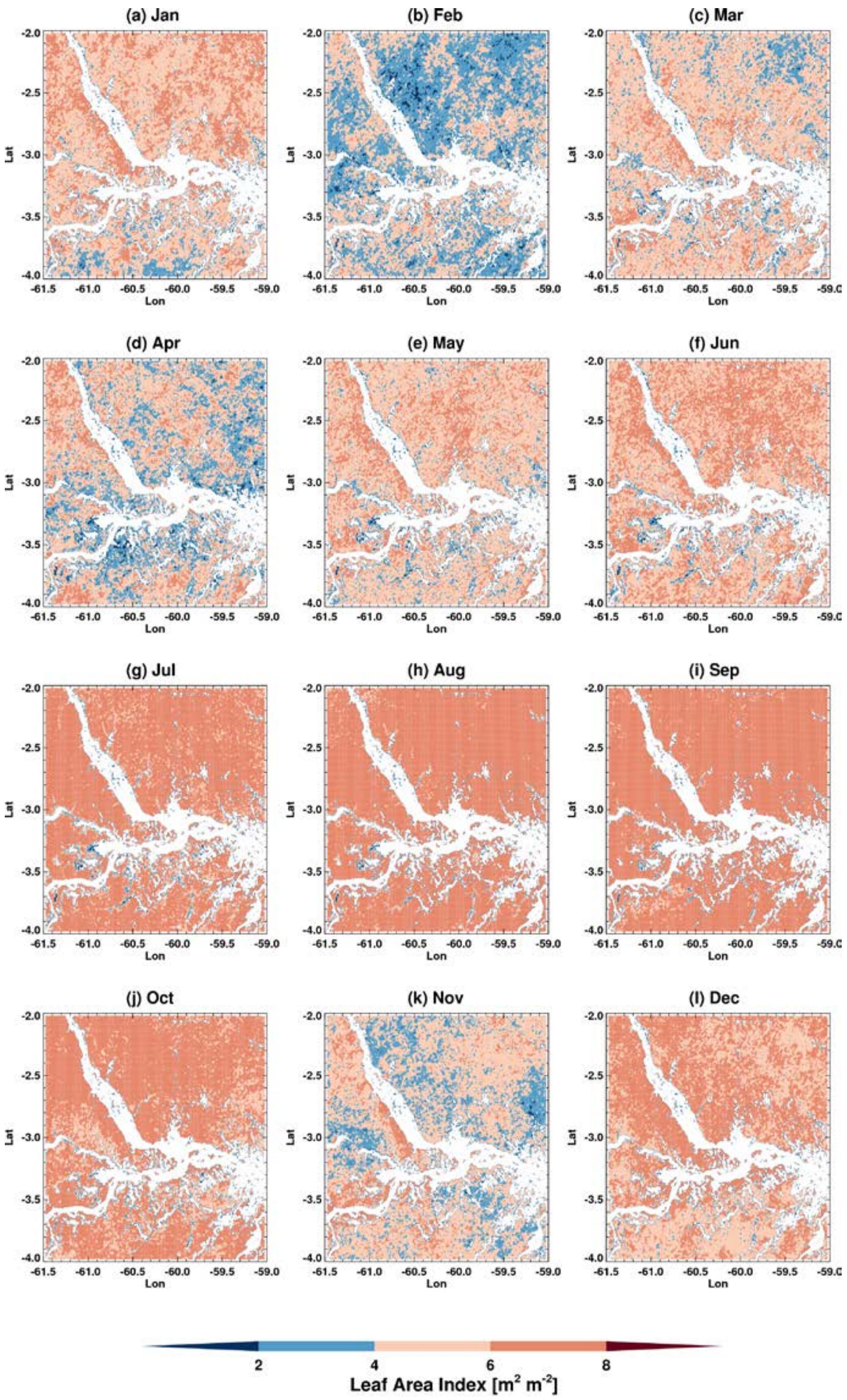

Supplementary Figure 4. Monthly LAI values. From (a) to (l) show the values of LAI from January to December during 2014 in the study domain from MODIS data.

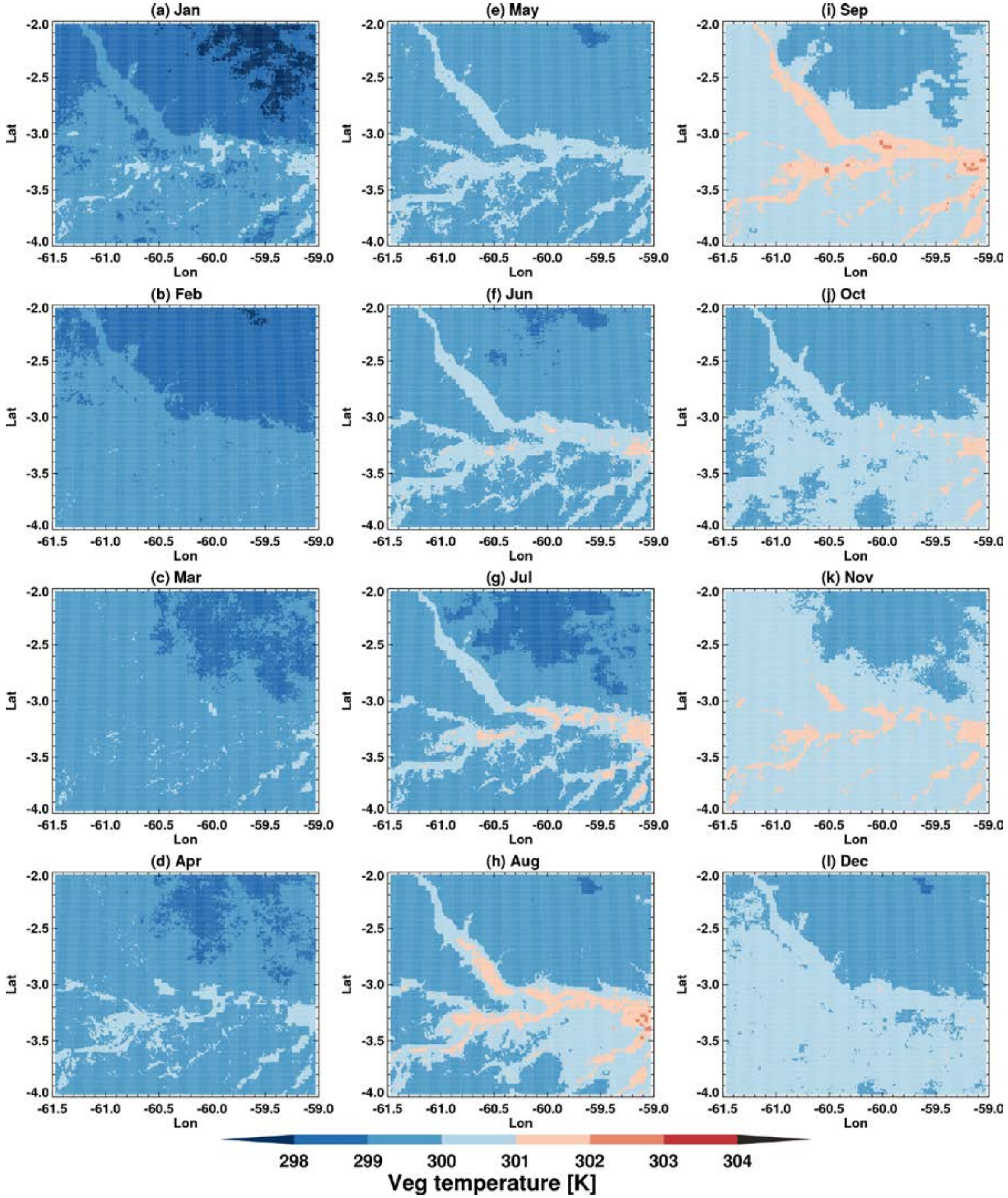

Supplementary Figure 5. **Simulated vegetation temperature.** From (a) to (l) show the vegetation temperatures from January to December during 2014 in the study domain simulated by CLM model.

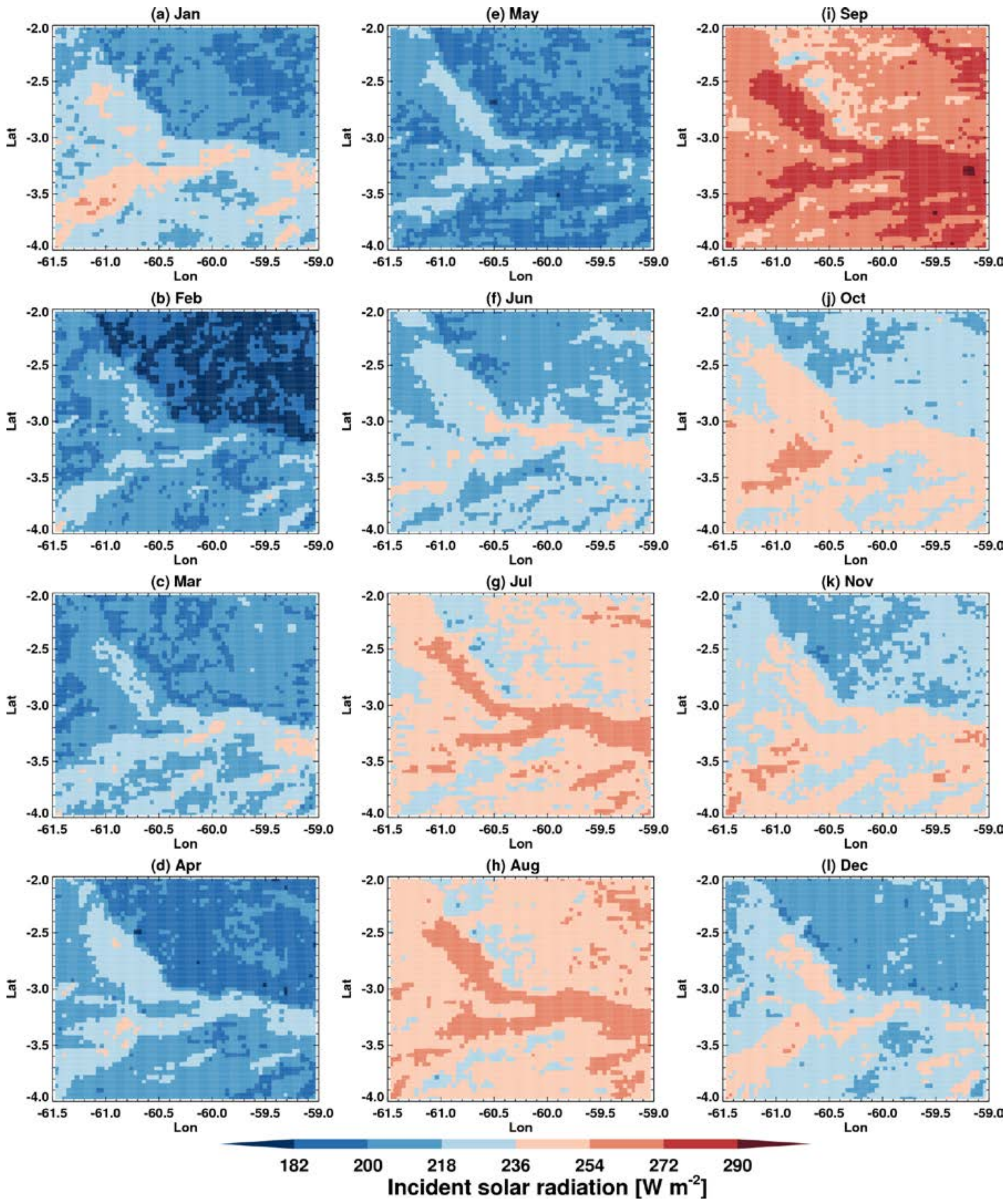

Supplementary Figure 6. **Simulated incident solar radiation.** From (a) to (l) show the incident solar radiations from January to December during 2014 in the study domain simulated by CLM model.

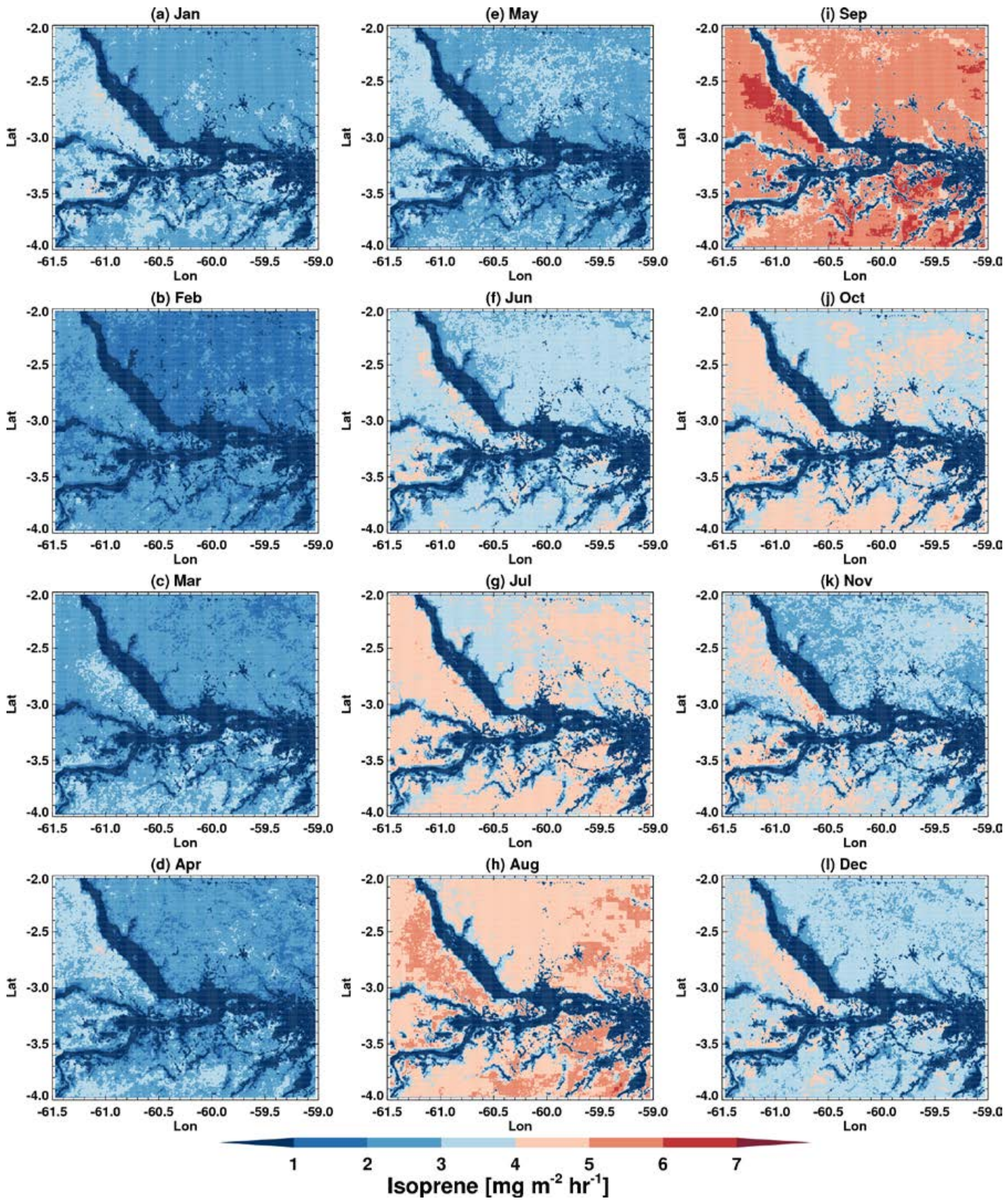

Supplementary Figure 7. **Simulated isoprene emission.** From (a) to (l) show the 24-hour monthly average isoprene emission rates from January to December during 2014 in the study domain simulated by CLM model.

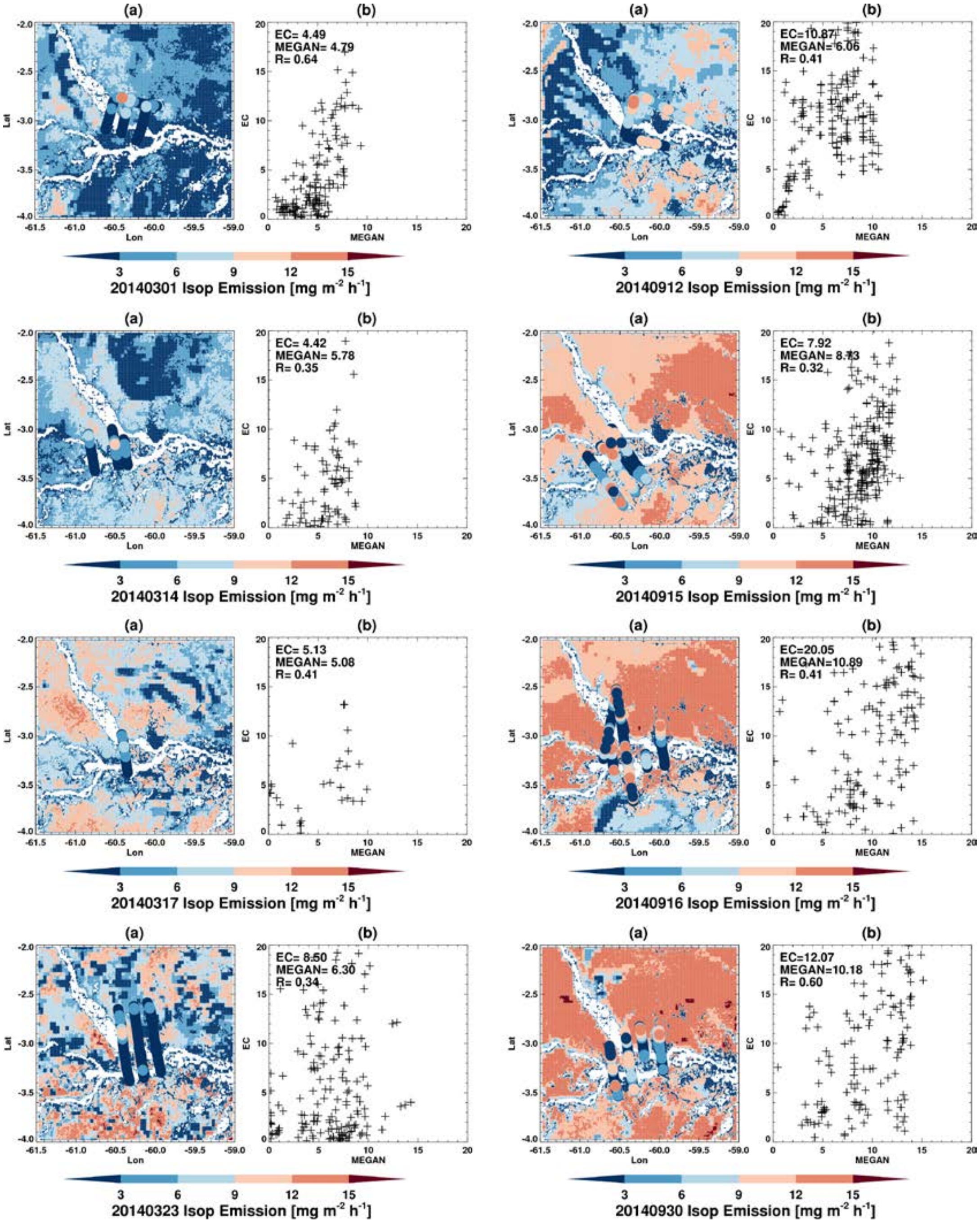

Supplementary Figure 8. **Surface isoprene emission flux during eight selected research flights.** (a): spatial distributions from airborne EC method (solid circles) compared with MEGAN simulations (background colors); (b): the mean values and linear correlation coefficient from the EC and MEGAN estimates in the scatter plot.

Supplementary Table 1. Isoprene EFs from EC and MEGAN estimates. Directly observed EFs from two dominant PFT, Broadleaf evergreen tropical tree and grass, from EC estimates and MEGAN model.

		Broadleaf evergreen tropical tree (mg m^{-2} h^{-1})	Grass (mg m^{-2} h^{-1})
Wet season	MEGAN EF	7	0.8
	EC EF	11.29	5.29
	Data number	418	3
Dry season	MEGAN EF	7	0.8
	EC EF	10.49	12.39
	Data number	848	4

Supplementary Table 2. Comparisons of Isoprene EFs from EC and MEGAN estimates with LAI. Basal EFs from EF estimates and MEGAN model compared with LAI with the interval of 1 m^2 m^{-2}.

LAI (m^2 m^{-2})	Wet season			Dry season		
	MEGAN EF (mg m^{-2} h^{-1})	EC EF (mg m^{-2} h^{-1})	Data number	MEGAN EF (mg m^{-2} h^{-1})	EC EF (mg m^{-2} h^{-1})	Data number
0-1	7	41	8	7	9.43	5
1-2	7	9.48	13	7	7.49	14
2-3	7	15.79	20	7	8.36	18
3-4	7	11.29	72	7	12.06	7
4-5	7	14.66	125	7	10.82	22
5-6	7	7.73	127	7	11.22	93
6-7	7	6.23	51	7	10.49	687
7-8	7	0	0	7	0	0
8-9	7	0	0	7	0	0
9-10	7	0	0	7	0	0
